# Label-Free Sequence-Specific Visualization of LAMP Amplified *Salmonella* via DNA Machine Produces G-Quadruplex DNAzyme

**DOI:** 10.3390/bios13050503

**Published:** 2023-04-26

**Authors:** Huan Zeng, Shuqin Huang, Yunong Chen, Minshi Chen, Kaiyu He, Caili Fu, Qiang Wang, Fang Zhang, Liu Wang, Xiahong Xu

**Affiliations:** 1 College of Biological Science and Engineering, Fuzhou University, Fuzhou 350108, China; 2 Institute of Agro-Product Safety and Nutrition, Zhejiang Academy of Agricultural Sciences, Hangzhou 310021, China; 3 Technology Center of Fuzhou Customs District, Fuzhou 350015, China; 4 Key Laboratory of Traceability for Agricultural Genetically Modified Organisms, Ministry of Agriculture and Rural Affairs, Hangzhou 310021, China

**Keywords:** loop-mediated isothermal amplification, colorimetric detection, sequence-specific, G-quadruplex, DNA machine

## Abstract

*Salmonella* is one of four key global causes of diarrhea, and in humans, it is generally contracted through the consumption of contaminated food. It is necessary to develop an accurate, simple, and rapid method to monitor *Salmonella* in the early phase. Herein, we developed a sequence-specific visualization method based on loop-mediated isothermal amplification (LAMP) for the detection of *Salmonella* in milk. With restriction endonuclease and nicking endonuclease, amplicons were produced into single-stranded triggers, which further promoted the generation of a G-quadruplex by a DNA machine. The G-quadruplex DNAzyme possesses peroxidase-like activity and catalyzes the color development of 2,2′-azino-di-(3-ethylbenzthiazoline sulfonic acid) (ABTS) as the readouts. The feasibility for real samples analysis was also confirmed with *Salmonella* spiked milk, and the sensitivity was 800 CFU/mL when observed with the naked eye. Using this method, the detection of *Salmonella* in milk can be completed within 1.5 h. Without the involvement of any sophisticated instrument, this specific colorimetric method can be a useful tool in resource-limited areas.

## 1. Introduction

Foodborne disease is a growing public health problem worldwide, usually contaminating food through any stage of food production, delivery, and consumption chain [1]. There are nearly 1 in 10 people around the world falls ill after eating contaminated food, which leads to over 420,000 deaths every year [2]. Salmonellosis is a common intestinal infection caused by *Salmonella* spp. *Salmonella* has a high detection rate in raw milk, cheese, raw meat, raw eggs, fruits, and vegetables [3], leading to diarrhea, vomiting, abdominal pain, chills, fever, headache, etc. People will get a foodborne illness when they eat undercooked meat or eat other foods or beverages that are contaminated by raw meat or its juices. However, as the “gold standard method”, the conventional culture method needs to be pre-accumulated, with selective separation and biochemical identification of the samples. It is accurate but time-consuming and very labor-intensive. It follows that it is necessary to control pathogen-based food poisoning outbreaks with an earlier, more rapid, and more sensitive method.

The nucleic acid test is a powerful technique for molecular diagnosis by analyzing the genetic sequence in organisms. Among them, isothermal amplification technology has drawn much attention because of the capability of on-site utilization. Compared to methods that require thermal cycling, isothermal amplification is performed at a single reaction temperature, so it is more rapid and more energy efficient. Loop-mediated isothermal amplification (LAMP) is one of the most promising and comprehensively applied isothermal amplification techniques. Developed by Tsugunori Notomi in 2000, LAMP is realized with four specially designed primers recognizing six distinct sequences on the target, which ensures its high specificity [4]. The cauliflower-like structured products possess abundant stem–loops that initiate the next cycle by hybridizing with the inner primer [4]. Therefore, the amplification can accumulate 10^9^ copies of the target in less than an hour. By introducing loop primers, its amplification efficiency can be further improved, and the amplification process can be accomplished in half-hour [5]. The amplification process can also be real-time monitored by collecting the fluorescent signal with an exclusive instrument [6], which permits its wide application for the detection of foodborne pathogenic bacteria [7,8], infectious diseases [9,10], and genetically modified organisms investigated by the artificial mouth simulator [11,12]. In order to make full use of LAMP in point-of-care diagnostic platforms, it is preferable to analyze the amplicons visually. With the visual method, the nucleic acid test can be achieved at point-of-care testing with high convenience in resource-poor settings because it does not rely on big and heavy instruments.

Recently, some ingenious colorimetric methods have been developed by detecting the generated amplicons or by monitoring the variation of reaction compositions [13]. For example, some intercalating dyes can bind double-stranded amplicons and indicate positive amplification by changing color [14]. The generation of pyrophosphate ions [15,16], the pH variation [17], and the consumption of deoxyribonucleotides (dNTPs) [18] in positive amplifications also enable colorimetric detection by the naked eye. However, how to visually detect the sequence-specific products of LAMP is still a great challenge.

G-quadruplex structures are composed of two or more stacked guanine (G)-tetrad planes and a monovalent cation such as K^+^ or Na^+^, which are formed at specific G-rich regions in the genome, mRNA, and non-coding RNA, and G-quadruplex DNAzymes are stacked G-tetrads structure with peroxidase-like activity when binding hemin (iron (III)-protoporphyrin IX) [19]. They can catalyze the color change of substrate, such as 2,2′-azino-di-(3-ethylbenzthiazoline sulfonic acid) (ABTS) and 3,3′,5,5′-Tetramethylbenzidine. Taking advantage of the catalytic activity of the G-quadruplex DNAzyme, colorimetric sensors can be developed for the analysis of DNA amplification [20,21,22].

DNA machine is constructed from DNA self-assembly depending on the sequence-specific interactions between complementary sequences [23]. The base sequence of nucleic acids encodes substantial structural and functional information into biopolymers [24], such as the base pairing, the pH-induced self-assembly of the C-rich sequence into i-motif configurations [25], and the ion-induced self-organization of the G-rich sequence into the G-quadruplex [26]. With rational design, DNA machines can perform machine-like functions by autonomously generating expected sequences in the presence of the appropriate trigger [27,28]. The powerful amplification ability exhibits great potential in constructing biosensors [29,30,31].

Here, by combining G-quadruplex DNAzymes and DNA machine, we present a sequence-specific method for colorimetric detection of LAMP amplicons. Typically, the LAMP amplicons are digested into short fragments by a restriction endonuclease. A nicking endonuclease recognition site is introduced into the inner primer to facilitate the generation of sequence-specific single-stranded amplicons by repeatedly nicking and extending. This restriction endonuclease and nicking endonuclease-mediated amplification are termed as LAMP-Res-Nick. The ssDNA products from the LAMP-Res-Nick reaction can trigger cascade amplification via DNA machine to generate G-quadruplex. The peroxidase-like activity of G-quadruplex DNAzymes, when binding hemin, makes the colorimetric readouts possible.

## 2. Materials and Methods

### 2.1. Reagents and Oligonucleotides

Bst DNA polymerase (Large Fragment) was purchased from Vazyme Biotech Co., Ltd. (Nanjing, China). Nt.BstNBI, ScrFI, DraI, and agarose were obtained from New England Biolabs (Ipswich, MA, USA). Syto 9 was achieved from Thermo Fisher (Waltham, MA, USA). 4-(2-Hydroxyethyl) piperazine-1-ethanesulfonic acid (HEPES), hemin, dNTP mixture, and ABTS were all supplied by Sangon Biotech (Shanghai, China) Co., Ltd. LB agar and LB broth were offered by Beijing Land Bridge Technology Co., Ltd. (Beijing, China) 30% H_2_O_2_ was bought from Sigma-Aldrich, Inc. (Burlington, MA, USA).

The gene of invasion protein A (invA) was selected as the reference gene for amplification (GenBank accession no. NC_003197). Conventional LAMP primers were synthesized according to the previous report [32]. Inner primers incorporated with the recognition site (GAGTC) of nicking endonuclease, Nt.BstNBI, are termed as Nick-BIP and Nick-FIP. All the sequences were evaluated with IDT Oligo Analyzer 3.1 (Integrated DNA Technologies, Coralville, IA, USA). The oligonucleotides were synthesized by Sangon Biotech (Shanghai, China) Co., Ltd. Sequences used in this work are listed in Table S1, and the corresponding template sequence is displayed in Figure S1.

### 2.2. DNA Extraction and Purification

The bacteria were separated by streak method on the LB agar plate, and a single colony was selected for further culturing in LB broth overnight.

DNA was extracted with the Bacteria Genome DNA Isolation kit (Spin Column) (Bioteke Corporation, Beijing, China) and stored at −20 °C. The concentration and purity of the extracted DNA were determined by NanoDrop One (Thermo Fisher, Waltham, MA, USA) for counting the copy number.

### 2.3. Real-Time LAMP Assay

For real-time LAMP, 10 μL amplification buffer contained 1 μL *Salmonella* DNA, 1.6 μM FIP and BIP, 0.2 μM F3 and B3, 0.4 μM LF and LB, 1.4 mM dNTPs, 3.2 U Bst DNA Polymerase (Large Fragment), 1× ThermoPol Reaction Buffer, 6 mM MgSO_4_, 1 mM SYTO 9. The amplification was performed on CFX 96 (Bio-Rad, Hercules, CA, USA) at 65 °C with fluorescence collected every 30 s. The products were analyzed with 3% agarose gel, and the gel results were recorded via gel image system (UVP, Upland, CA, USA).

### 2.4. Cascade Amplification of LAMP-Res-Nick and DNA Machine

For LAMP process, the inner primer FIP was replaced by Nick-FIP, and no Syto 9 was involved. The reaction was performed at 65 °C on an MSC-100 ThermoMixer (AllSheng, Hangzhou, China) for 20 min as the protocol described in Section 2.4. A total of 20 μL solution contained 10 μL of LAMP products, 50 mM Tris-HCl (pH 7.9), 100 mM NaCl, 6 mM MgSO_4_, 100 μg/mL BSA, 10 U ScrFI. The reaction was incubated at 37 °C for 1 h followed at 95 °C for 10 min. Then, 1.5 μL 10× Isothermal Amplification Buffer II Pack, 4.8 U Bst 3.0 DNA Polymerase, 10 U Nt.BstNBI, 0.32 mM dNTPs were added to make the solution 25 μL. The reaction was performed at 58.8 °C for 10 min. Thereafter, 5 μL of 1 μM M-G was added, and the mixture was incubated at 58.8 °C for another 10 min. The final products were incubated at 95 °C for 5 min followed on ice for 10 min.

### 2.5. Colorimetric Detection by G-Quadruplex DNAzyme

For colorimetric detection, the cascade amplification products were mixed with 1× HEPES buffer (25 mM HEPES, 20 mM KCl, 200 nM NaCl, and 0.05% Triton X-100, pH 5.3), 200 nM hemin, and 1 μL of 1M HCl. Then 2 mM ABTS^2−^ and 2 mM H_2_O_2_ were added in the final 100 μL system for naked-eye detection in 5 min. The RGB values were extracted by Image J.

### 2.6. Sensitivity and Specificity

In order to test the sensitivity, 1 μL of a series of 10-fold diluted DNA was employed as template. To test the specificity of the method, DNA extracted from *Salmonella* typhimurium CMCC(B)50115, Vibrio parahemolyticus KP9, Vibrio parahemolyticus ATCC 17802 and Escherichia fergusonii 19ZEF91003 were employed as the templates. DNA was amplified and colorimetrically detected by the protocol described.

### 2.7. Detection of Salmonella Spiked Milk

Select *Salmonella* typhimurium colony and transfer it into LB broth for culturing at 37 °C for 6 h. The bacteria solution was then diluted 10-fold with saline and plate cultured for counting the colony number. The diluted solution was spiked into sterile milk with 10 times dilution. *Salmonella* DNA was extracted by the Bacteria Genome DNA Isolation kit. A total of 1 μL of extracted DNA was amplified and detected by the method described. All experiments were repeated 3 times. Results were shown as mean ± standard deviation. Differences were assessed by ANOVA.

## 3. Results and Discussion

### 3.1. Proof of Principle

**Scheme 1:**
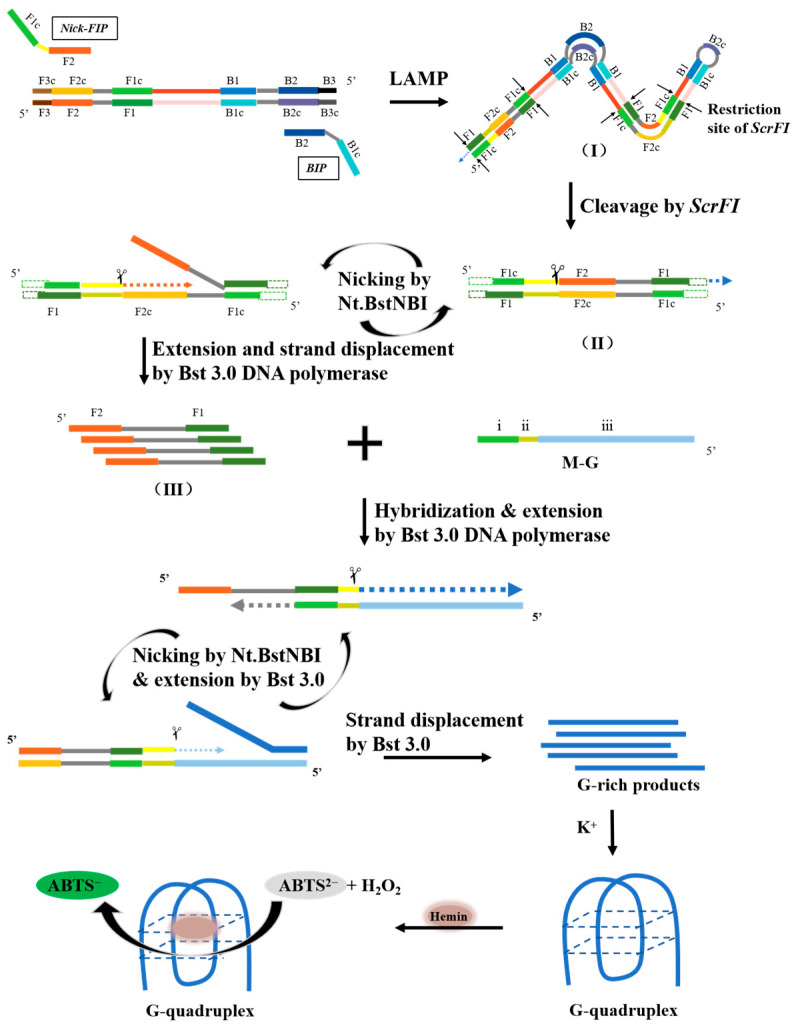
Schematic illustration of the colorimetric and sequence-specific method for detection of LAMP products via DNA machine and G-quadruplex DNAzyme.

In this study, the colorimetric and sequence-specific method is realized by producing a G-quadruplex DNAzyme by the cascade amplification of restriction endonuclease- and nicking endonuclease-mediated LAMP (LAMP-Res-Nick) and DNA machine. As shown in Scheme 1, the recognition site of nicking endonuclease is incorporated in the inner primer (Nick-FIP) and acts as the spacer between F1c and F2. The LAMP process produces a great variety of stem–loop DNAs (I). These DNAs, which have different stem lengths and possess multiple loops, provide a great number of restriction endonuclease recognition sites. Thanks to the restriction endonuclease, these products of different structures are cleaved into short double-helix fragments with the nicking endonuclease recognition sites embedded in them. Thereafter, nicking endonuclease, Nt.BstNBI, will recognize these sites and produce a nick on the double-stranded products. Meanwhile, Bst polymerase will add new free nucleotides at the 3′ end of the nicking site and displace the original strands. The synergistic effect of Bst polymerase and Nt.BstNBI promotes repeatedly nicking and extension that innumerable single-stranded products are generated (III). In order to transform this DNA sequence information into color development, a DNA machine (M-G) is added to realize cascade amplification. The M-G compromises three parts, i.e., a complementary sequence of the generated ssDNA products at the 3′ terminus, a nicking endonuclease recognition site in the middle, and a C-rich sequence at the 5′ terminus. Once the ssDNAs hybridize with the DNA machine, a great deal of G-rich sequences that can form G-quadruplex structures with the presence of potassium ions are produced. The complex, formed by hemin binding to the G quadruplex, possesses the peroxidase-mimicking activity, which can catalyze the oxidation of the substrate ATBS^2−^ to ATBS^−^ by H_2_O_2_, thereby turning the solution green. On the contrary, in the absence of targets, the LAMP process cannot be initiated, and the G-rich sequence cannot be produced. Consequently, no color change is observed.

### 3.2. Effect of Nicking Endonuclease Recognition Site

**Figure 1:**
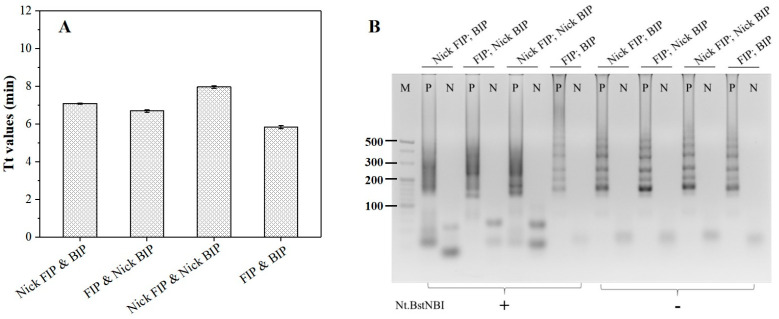
Investigate the effect of inner primers incorporated with a nicking endonuclease recognition site (termed as Nick-FIP and Nick-BIP) on LAMP process by (**A**) Tt values of real-time LAMP and (**B**) gel electrophoresis. Nt.BstNBI was added to incubate with the LAMP products for 10 min. N stands for no template control, P stands for positive control with 10^5^ copies of *Salmonella* DNA, and M stands for 20 bp DNA ladder.

The recognition site (GAGTC) of nicking endonuclease, Nt.BstNBI, was inserted in the inner primers (termed Nick-FIP and Nick-BIP). We used only one or two modified inner primers for real-time LAMP and compared them with conventional inner primers. As shown in Figure 1A, compared with conventional inner primers, the incorporation of a nicking endonuclease recognition site in the inner primer reduced the amplification efficiency. However, the taking off time only delayed 2 min. The amplification was further verified by gel electrophoresis (Figure 1B). All these positive amplifications, including the inner primers incorporated with a nicking endonuclease recognition site, generated ladder-like bands. In contrast, no obvious non-specific products were produced in all of the negative samples. These results indicate that the presence of a nicking endonuclease recognition site in the inner primer has little effect on the LAMP process. To prove the nicking endonuclease recognition site was successfully incorporated in the LAMP products, the products were incubated with the nicking endonuclease, Nt.BstNBI, for another 10 min. As shown in the electrophoresis image (Figure 1B), the ladder-like bands became smeared when the modified inner primers were used for amplification. In contrast, the products produced by the conventional primers were still ladder-like. This indicates the successful incorporation of the nicking endonuclease recognition site into the LAMP amplicons. Since only one modified inner primer was enough for introducing the nicking endonuclease recognition site into the LAMP products, we used modified FIP for the subsequent experiments.

### 3.3. Effect of Restriction Endonuclease

**Figure 2:**
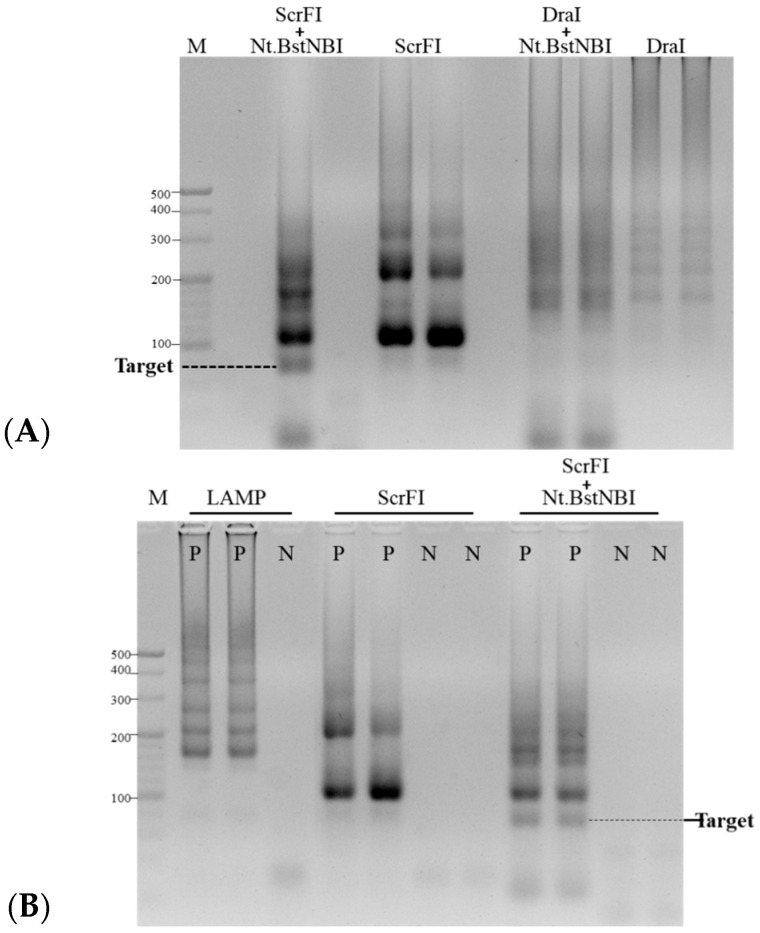
(**A**) In the presence of 10^5^ copies of Salmonella DNA, LAMP products were cleaved by restriction endonucleases ScrFI and DraI, respectively, followed by treatment with nicking endonucleases Nt.BstNBI, then electrophoresis in 3% agarose gel. (**B**) 3% agarose gel electrophoresis showing variability in products generated after isothermal amplification and these products cleaved by ScrFI and Nt.BstNBI, N means no template control, N stands for no template control, P stands for positive control with 10^5^ copies of Salmonella DNA, and M stands for 20 bp DNA ladder.

According to the previous study, the nicking and extension process prefers producing short single-stranded DNA sequences. Since the LAMP products are cauliflower-like structures with multiple loops, they can provide multiple nicking sites. Therefore, the single-stranded DNA products are of different lengths because of the strong processivity of Bst polymerase and random nicking by Nt.BstNBI. We employed restriction endonuclease, ScrFI, and DraI, respectively, to cleave the LAMP products into small fragments which contained the same 3′ terminus. The restriction sites of ScrFI and DraI are illustrated in Figure S2A, and the expected cleaved products are indicated in Figure S2B. As shown in Figure 2, after digestion by ScrFI, the ladder-like bands disappeared. Instead, the products were enriched at around 100 bp and 200 bp, indicating the products were cleaved by ScrFI. By contrast, when the products were treated with DraI, the products were still ladder-like bands on the gel; this might be attributed to the poor compatibility of buffer for DraI and LAMP.

To further verify the cleavage of LAMP products by ScrFI, we added Nt.BstNBI into the system and observed the generation of 73 bp products (Figure 2). This was attributed to the synergistic effect of Bst polymerase and Nt.BstNBI, which promoted repeated nicking and extension from the nicking site on the LAMP products and displaced the sequence downstream of the nicking site. In contrast, when Nt.BstNBI was added to the samples treated by DraI, and the ladder-like bands became smeared. This was probably owing to the LAMP products not being digested before nicking, and the dissociated single-stranded sequence being of different lengths.

Based on these results, ScrFI was used for cleaving the LAMP products into short fragments in the following experiment.

### 3.4. Sensitivity

**Figure 3:**
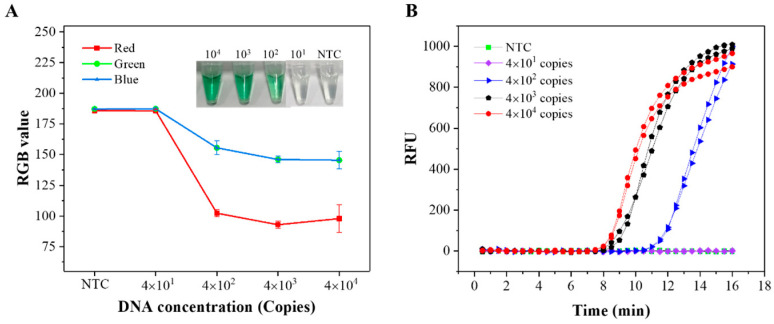
(**A**) RGB values of colorimetric assay of different concentrations of DNA (inset is the colorimetric photo). (**B**) Real-time LAMP assay of different concentrations of DNA. NTC indicates no template control. Error bars indicate standard deviations for *n* = 3.

It is reported that *Salmonella* is one of the four key reasons that cause diarrhea diseases [33]. Around 3.4 million cases of diseases are caused by invasive nontyphoidal *Salmonella* annually [31]. To test the feasibility of the developed method, we chose *Salmonella* typhimurium as an example. DNA extracted from *Salmonella* was serially diluted as 4 × 10^1^, 4 × 10^2^, 4 × 10^3^, and 4 × 10^4^ copies/μL for sensitivity evaluation, and the template was amplified by LAMP-Res-Nick and DNA machine. The products were evaluated by G-quadruplex DNAzyme catalyzing the color development of ABTS. Since the e RGB pattern can effectively eliminate the error in human observation, the RGB value was also extracted and analyzed, and the RGB values were extracted by Image J. The RGB (red, green, and blue) is an important index for color expression. Each channel of red, green, and blue has 256 levels of brightness, of which level 0 means the darkest and 255, the brightest. The results are shown in Figure 3A. There was no color change in the negative control and the sample with 4 × 10^1^ copies of DNA. In contrast, an obvious green was developed for samples containing 4 × 10^2^, 4 × 10^3^, and 4 × 10^4^ copies of the template. The green and blue channels were a little more sensitive, which dropped from color density 184 (without target) to 148 (4 × 10^3^ copies/μL target), and the best sensitivity was achieved for the red channel as color density dropped from 184 to 94. Therefore, the red channel was chosen for further assays as the optimal one. The results were compared with real-time LAMP (Figure 3B), and consistent results were obtained.

### 3.5. Specificity

**Figure 4:**
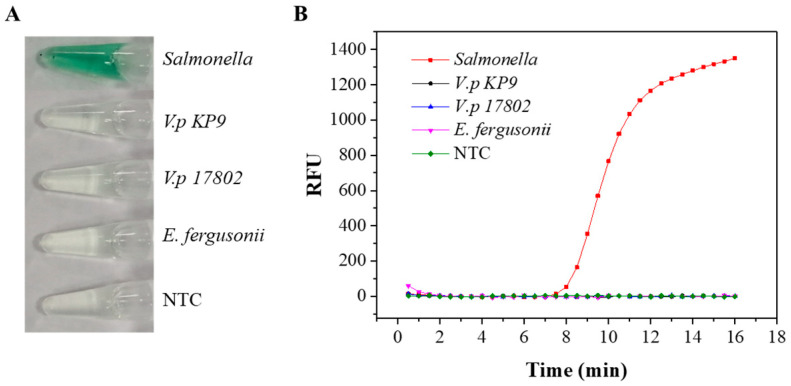
Specificity of (**A**) colorimetric assay and (**B**) real-time LAMP. The amount of *Salmonella* and nontarget strains (*V.p KP9*, *V.p 17802*, and *E. fergusonii*) were 10^3^ copies, and no DNA template was used as NTC.

To test the specificity of the method, DNA extracted from *Salmonella* typhimurium CMCC(B)50115, Vibrio parahemolyticus KP9, Vibrio parahemolyticus ATCC 17802, and Escherichia fergusonii 19ZEF91003 was detected. As shown in Figure 4A, only DNA extracted from Salmonella typhimurium developed green. In contrast, other samples gave colorless results. The results were consistent with real-time LAMP (Figure 4B).

### 3.6. Detection of Salmonella in Milk

**Figure 5:**
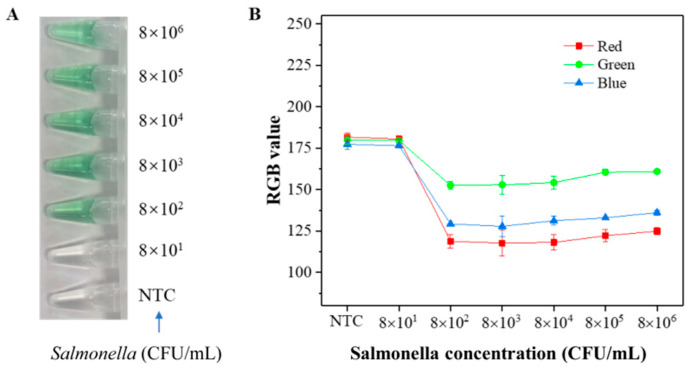
(**A**) Colorimetric detection of milk samples spiked with a series of concentrations of *Salmonella* typhimurium. (**B**) RGB values of the colorimetric assay. NTC indicates no template control. Error bars indicate standard deviations for *n* = 3.

*Salmonella* is one of the main pathogens in raw milk, and healthy people of any age can be gravelly sick after drinking raw milk contaminated with *Salmonella*, not only these people with weakened immune systems. From 2013 to 2018, there are 75 outbreaks reported to CDC were linked to raw milk, which included 675 illnesses and 98 hospitalizations [34]. Therefore, it is meaningful to test the practicability of the method for the detection of *Salmonella* contamination in milk. Here, milk samples spiked with a series of concentrations of *Salmonella* typhimurium, ranging from 8 × 10^6^ CFU/mL to 8 × 10^1^ CFU/mL, were detected. As shown in Figure 5A, milk contaminated by 800 CFU/mL or more of *Salmonella* turned green. As shown in Figure 5B, when the *Salmonella* concentration was lower than 800 CFU/mL, the red channel was close to 180. When increasing the *Salmonella* concentration made, the value of the red channel decreased significantly, indicating the sensitivity for *Salmonella* detection in spiked milk was 800 CFU/mL. The results confirm that our sensor can be used for practical sample analysis. Though this method does not improve the sensitivity (Table 1), the method does not require an exclusive instrument, and the colorimetric results can ensure specificity.

## 4. Conclusions

In summary, the key issue to realizing colorimetric and sequence-specific detection of LAMP amplicons is to efficiently transduce the amplification signal into color development of ABTS. In this work, we employed a cascade amplification of restriction endonuclease- and nicking endonuclease-mediated LAMP (LAMP-Res-Nick) and DNA machine to generate G-quadruplex and employed the peroxidase-mimicking activity of G-quadruplex DNAzyme to catalyze color development. The sensitivity was comparable to real-time LAMP. The specificity was confirmed by testing four kinds of common foodborne bacteria. The method was verified to be feasible for the detection of 800 CFU/mL *Salmonella* spiked milk by the naked eye. The biggest advantage of this method is that sequence-specific colorimetric readouts can be obtained simply, with no sophisticated instrument required during the whole process, which is promising for point-of-care utilization.

## Figures and Tables

**Table 1:** Comparison of the performance of our colorimetric method with other reported LAMP-based methods.

Method	Instrument or Device	Sensitivity	Results Determination	Method to Verify the Specificity	References
qLAMP	qPCR thermocycler	4 CFU/25 g (chicken)	Real-time fluorescence	Melting curve	[35]
LAMP-Turbidity	Real-time turbidimeter	6.1 × 10^3^–6.1 × 10^4^ CFU/g	Real-time turbidity	Agarose gel electrophoresis	[36]
LAMP-ELISA	A thermal cycler/water bath, a plater reader	10^3^ CFU/mL (spiked meat sample)	Absorbance	Capture the amplicons with specific probes and detect by ELISA	[37]
In situ LAMP	A water bath, a fluorescence microscope	1 CFU/cm^2^ (eggshells)	Microscopy	fluorescence microscope	[38]
Triplex LAMP	Genie III LAMP detector	64 CFU/g (chicken meat)	Real-time fluorescence	Melting curve	[39]
LAMP on a microfluidic compact disc	a digital RPM meter, a spinning motor, an IR thermometer	3.4 × 10^4^ CFU/mL (spiked tomato)	Visual observation	N^a^	[40]
Real-time LAMP	Genie III LAMP detector	1.2–12 CFU/reaction	Real-time fluorescence	N^a^	[41]
LAMP on a chip	an eight-channel pump, a heater, and a small ESE log detector	50 cells/test (pork meat)	Real-time fluorescence	N^a^	[42]
Microfluidic LAMP	A rotary system consists of three heating blocks, a servo motor	50 CFU/mL (tap water or milk)	lateral flow strip	Capture the amplicons with antibody	[43]
Visual LAMP	A metal heater	800 CFU/mL for milk sample	Visual observation	DNA machine for transferring the target sequence to DNAzyme	This manuscript

N^a^: Not mentioned in the article.

## Data Availability

Data are contained within the article or supplementary material.

## Supplementary Materials

The following supporting information can be downloaded at: https://www.mdpi.com/article/10.3390/bios13050503/s1, Table S1: Sequences employed in this work.; Figure S1: Template sequence for LAMP-Res-Nick amplification of the invA gene of *Salmonella*; Figure S2: The effect of restriction endonuclease, ScrFI, and DraI, on cleaving LAMP products of invA gene of *Salmonella*. (A) Scheme illustrating the restriction sites of ScrFI and DraI on the long stem–loop structure of LAMP products. Red arrows indicate the restriction site of ScrFI, and the blue arrows indicate the restriction site of DraI. (B) The expected LAMP products extended from Nick-FIP. The numbers in the box denote the expected length of the amplification products.
