# Supplementary material for: Label-Free Sequence-Specific Visualization of LAMP Amplified Salmonella via DNA Machine Produces G-Quadruplex DNAzyme
Source: Biosensors (Basel). 2023 Apr 26;13(5):503. doi: 10.3390/bios13050503 (PMC10216017; doi:10.3390/bios13050503)
Supplement: Supplementary file 1 [file biosensors-13-00503-s001.zip › biosensors-2302243-supplementary.pdf]

biosensors

MDPI

Supporting Information

Label-Free Sequence-Specific Visualization of LAMP Amplified *Salmonella* via DNA Machine Produces G-Quadruplex DNAzyme

Huan Zeng [1], Shuqin Huang [1], Yunong Chen [1,2], Minshi Chen [3], Kaiyu He [2], Caili Fu [1], Qiang Wang [2], Fang Zhang [1,*], Liu Wang [2,4,*] and Xiahong Xu [2]

[1] College of Biological Science and Engineering, Fuzhou University, Fuzhou 350108, China; 210820068@fzu.edu.cn (H.Z.); 200820041@fzu.edu.cn (S.H.); yunong0420@163.com (Y.C.); caili_fu@hotmail.com (C.F.)
[2] Institute of Agro-product Safety and Nutrition, Zhejiang Academy of Agricultural Sciences, Hangzhou 310021, China; hekaiyu@zaas.ac.cn (K.H.); wq13575733860@126.com (Q.W.); xuxiahong@zaas.ac.cn (X.X.)
[3] Technology Center of Fuzhou Customs, Fuzhou 350015, China; minshi0271@sina.com
[4] Key Laboratory of Traceability for Agricultural Genetically Modified Organisms, Ministry of Agricul-ture and Rural Affairs, Hangzhou 310021, China
* Correspondence: fangzh921@fzu.edu.cn or zhangfang921@gmail.com (F.Z.); wangliually@126.com (L.W.)

1. Supplementary Tables and Figures

1.1. Supplementary Tables

Table S1. Sequences employed in this work.

Oligonucleotides [a]	Sequence (5′-3′) [b,c,d]
FIP	TCCCGGCAGAGTTCCCATTGAAATCATGAC-GCAGCTGTTGAA
BIP	TTCCCGCTGCCGGTATTTGTTGCTACGTTTTGCTTCAC-GGA
F3	GCGATAATATGGGGCGGAAT
B3	CGCCTTTGCTGGTTTTAGGT
LF	TATTCGGTGGTTTTAAGCGTACTC
LB	GCCGTAACAACCAATACAAATGG
Nick-FIP	TCCCGGCAGAGTTCCCATTGAAGAGTCATCATGAC-GCAGCTGTTGAA
Nick-BIP	TTCCCGCTGCCGGTATTTGTTGAGTCGCTACGTTTT-GCTTCACGGA
M-G	**TCCCAACCCGCCCTACCC**TTTT*GACTC*GGCAGAG-TTCCCATTGAAAT

[a] FIP and BIP are inner primers. F3 and B3 are outer primers. LF and LB are loop primers. Nick-FIP and Nick BIP are FIP and BIP, respectively, incorporated with a nicking endonuclease recognition site. M-G is the molecular machine. [b] Underlined letters indicate the recognition site of Nt.BstNBI. [c] Italic letters in bold indicate the complementary bases of the recognition site of Nt.BstNBI. [d] Under-lined letters in bold indicate the complementary sequence of the G-rich sequence.

1.2. Supplementary Figures

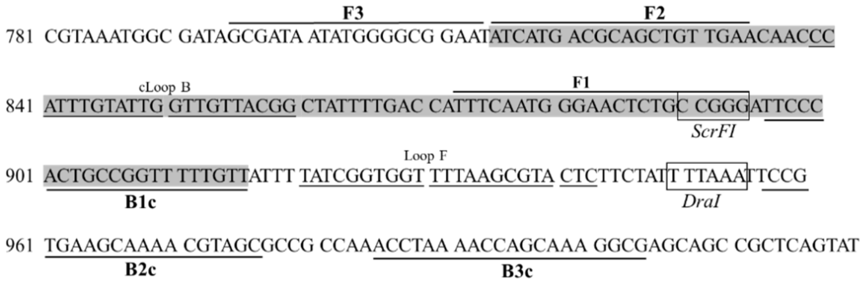

Figure S1. Template sequence for LAMP-Res-Nick amplification of the invA gene of *Salmonella*.

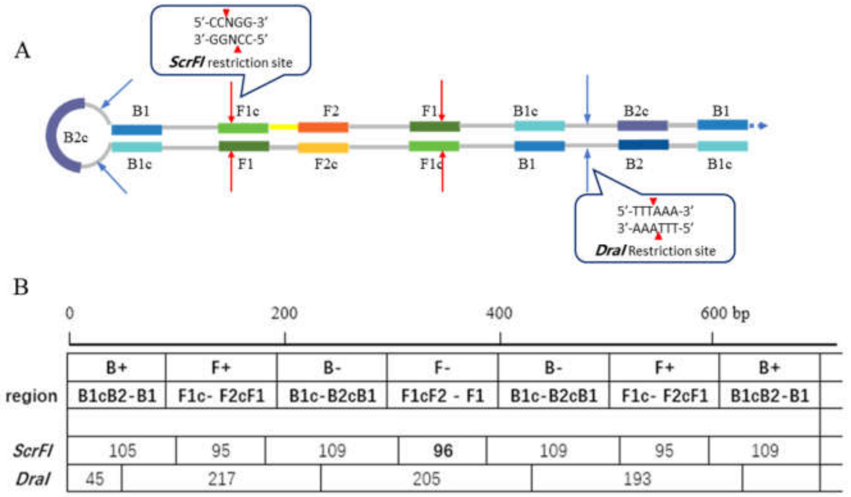

Figure S2. The effect of restriction endonuclease, ScrFI, and DraI, on cleaving LAMP products of invA gene of *Salmonella*. (**A**) Scheme illustrating the restriction sites of ScrFI and DraI on the long stem-loop structure of LAMP products. Red arrows indicate the restriction site of ScrFI. And the blue arrows indicate the restriction site of DraI. (**B**) The expected LAMP products extended from Nick-FIP. The numbers in the box denote the expected length of the amplification products.
